# Chronic Catarrh of the Middle Ear.—III

**Published:** 1894-09-08

**Authors:** 


### MEDICAL PROGRESS AND HOSPITAL CLINICS.

### CHRONIC CATARRH OF THE MIDDLE EAR.—III.

**Diagnosis.**—From what has been said regarding the clinical history of chronic middle ear catarrh, we trust sufficient will have been gathered to enable our readers to distinguish for themselves the chronic from the acute and subacute forms of aural inflammation. The subject of diagnosis will be most advantageously pursued, by describing the best mode of investigating a case when it comes before us. The question now is one of working out details; we may know that we are dealing with a middle ear catarrh, but we must find out whether it is of recent date and favourable in character, whether there is evidence of the presence or absence of fluid secretions, and whether, some degree of deafness being evidently established, to what extent this is the case. If the deafness is manifestly of quite old standing, we must endeavour to ascertain with the help of such means as are at our disposal, whether structural changes have taken place in the sound-conducting organs, and where there is any involvement of the auditory nerve or labyrinth. It has already been observed that in sclerotic forms of catarrh, the labyrinth is undoubtedly implicated by continuity through the stapedio-vestibular articulation. It is, however, Politzer's opinion that this is not necessarily always the case, the labyrinth on the contrary, being occasionally the initial seat of disease, or being attacked, at least coincidently with the middle ear. Again it is probable that the nerve suffers in many old standing catarrhs from want of use, analogously to the amblyopia of an eye affected with strabismus, in which the image has long been disregarded.

A knowledge of these facts is important when we have to answer the question the patient is sure to ask, *i.e.*, whether there is any hope of doing him good. A cautious prognosis with reference to the complete restoration of hearing, unless the result of inflation is very marked, must always be given, however slight the degree of deafness may be.

The presence of subjective noises in the ear, whether pulsating or continuous, is rather an unfavourable symptom unless removed by inflation.

**Method of Examining the Patient.**—We ascertain how long he has been deaf, and whether the deafness came on suddenly or insidiously. In the former case we usually suspect cerumen. Unfortunately it is a common experience to find after removing a plug of wax, that some deafness still remains, due to an old standing catarrh, chronic middle ear inflammation and ceruminal accumulations being frequently associated. Elderly people who are getting very hard of hearing often suffer their meati to be completely blocked with cerumen without being conscious of the fact. It is well to make a cursory examination of the meatus externus while getting out the history in order to be able to exclude impaction of wax, exostoses, a foreign body, or suppurative disease, and exclude them from the diagnosis at once. We should inquire next as to the supposed cause of deafness, and as to the presence of tinnitus and vertigo; also as to variations in the degree of deafness. In the secretive forms of catarrh there are considerable fluctuations in the hearing power, and audition is rendered worse on catching a fresh cold, whereas in primary sclerosis the deafness is generally pretty constant, or if anything improved during a "cold in the head."

With reference to previous illness, it may be here mentioned that the aural sequela of influenza is generally one of the acute forms of otitis media; less frequently, it is followed by a labyrinthine lesion.

Acquired or congenital syphilis may complicate middle ear catarrh, or may primarily attack the labyrinth. The acquired form usually occurs as a late secondary or early tertiary manifestation; and it is important to note that the deafness which is generally bilateral, complete, and accompanied by tinnitus, comes on very rapidly or even suddenly. A general survey should be taken of the patient's health, especially with reference to anæmia, and the plethoric and gouty habits. Careful inspection of the teeth should never be omitted, since caries of these organs is often responsible reflexly for noises in the ear and for laying the foundations of chronic middle ear deafness, besides operating prejudicially on the general health by creating dyspeptic troubles.

**Testing the Hearing Power. Air Conduction.**—The next point is to test the hearing power of the patient, as measured by the voice, the watch, and the tuning fork, and the reader is advised to make it his invariable rule to jot down in his note-book the results of the tests, in the order in which the observations are made, so as to ascertain precisely what impression, beneficial or otherwise, is made upon the deafness by inflation and subsequent procedures. The hearing power for conversation is so important that we are always glad if we can improve a defect in that direction in the slightest degree. In estimating it, a very good practical suggestion of Dundas Grant's is to ascertain whether at a distance, say, of two yards, the patient can hear the voice when shouting, spoken loudly, in the usual tone, or whispered, If whispering is not heard at that distance, we approach the ear gradually until it becomes audible. In no case should the patient be allowed to see the movements of the lips.

The watch we carry about with us is naturally one of the first hearing tests we have recourse to, as our knowledge of the distance at which it ought to be heard by the normal ear, compared with that at which it has been heard by the deaf patients for whom we have used it, gives the results attained a definite value in our own minds. In order, however, that other people may be able to correctly interpret our observations, we must have some means of indicating the strength of the watch-tick and the patient's capacity for hearing it, coincidently; and this is done, somewhat as in testing sight, by means of a fraction. Thus H.P. (W.)=$\frac{6}{50}$ means that a watch which should be heard at a distance of fifty inches is heard at six, the denominator of the fraction representing the normal hearing distance for the watch, and the numerator the farthest distance at which it is audible to the patient. We shall always find a great deal of variation as regards these tests amongst deaf people, some hearing the watch well, but conversation badly, and *vice versâ*. A deficient appreciation of the watch tick is generally believed to point towards nerve involvement; whereas conversation is better heard in nerve deafness than in simple chronic middle ear catarrh. It is not advisable to measure the hearing with a watch having too loud a tick, as in that case it is difficult to estimate slight degrees of deafness with it. Fifty to sixty inches is about the average distance for hearing an English lever, and it should be brought from outside the range gradually nearer, but patients often ask to be allowed to hear it first in order to become familiar with the sound. If the watch is not detected when brought into gentle contact with the ear, we note down H P = — C. By employing pressure we learn nothing further differentially with respect to the conducting organs, as we are then transmitting vibrations through the tissues (tissue conduction), and this latter is better estimated by means of the tuning fork. The patient's hearing power for high and low tones is estimated by means of graduated tuning forks, ranging from C to $C^4$ or $C^5$, held near the meatus whilst vibrating. As a general rule, all low tones and conversation are better heard in nerve deafness; high pitched ones and the watch tick in middle ear, or obstructive deafness; but mixed cases are frequent. For testing the perception of the higher tones, above the scale of the tuning forks, Galton's whistle is employed.

**Bone Conduction.**—The next point is the examination of the bone conduction, in other words testing the integrity of the sound perceiving organs. For this purpose, a Gardiner Brown tuning fork, which is tuned to middle C, answers best. Set it vibrating by striking it gently on the knee, and ascertain first how long it is heard at the meatus, noting down any deficiency that exists in seconds (T.F. at meatus = — x″). This is of course a measure of the hindrance to aerial conduction by the middle ear of the vibrations of the tuning fork, but it is an advisable preliminary in a systematic examination. Next we place the foot of the vibrating fork firmly on the patient's mastoid, and ask him to tell us when he ceases to hear it, at which moment we transfer it to our own. If the case is one in which the conducting organs are alone involved, as in uncomplicated middle ear catarrh, we shall not hear it again on our own mastoid process, and this result will, in fact, be anticipated by a cessation of all feeling of vibrations by our fingers, before the patient ceases to hear them (Gardiner Brown.) This depends on the mportant fact that in middle ear catarrh (not in the impaction of wax), the vibrations are heard longer on the mastoid of the affected, than on that of the normal ear, bone conduction being increased (+). To measure the difference in such a case, we place the tuning fork on our own mastoid, and transfer it as soon as we cease to hear it to the patient's, crediting him with the result, thus (T.F. on mastoid = + x.) Should the case have been one of nerve deafness, bone conduction would of course have been diminished instead of increased. There are two other tests to be described, which yield confirmatory evidence as to the state of the sound perceiving and conducting organs respectively. The first is that of Weber; the vibrating fork is placed on the vertex, and the patient is asked on which side he hears it more distinctly. The rule is to hear it louder on the side on which there is some hindrance to sound conduction, as any one may prove for himself by gently stopping up one ear with the finger. If the fork is heard better on the deaf side, Weber's test is positive; if worse on the deaf side it is negative. The other test is Rinné's, and it is founded on the following experiment: Placing a vibrating tuning-fork on the normal mastoid, and transferring it as soon as it ceases to be audible, to the meatus, it is heard again for a few seconds. This is Rinné positive, or (+R); failing to be thus heard at the meatus, the test is negative (—R), and points, like Weber positive to obstruction in the conducting organs. Thus it will be seen that these two tests are in a manner related, for Rinné negative, and Weber positive, both depend upon increase of bone conduction, and indicate middle ear trouble. But as the higher tones are heard best in middle ear catarrh, a positive Rinné does not exclude slight degrees of catarrh unless it is positive for the lower ones. Rinné's test is negative, even for the higher tones in the majority of cases that come before us.

These tests are not absolutely infallible, so that some caution should be exercised in drawing conclusions from one alone, until the results obtained from it are confirmed by the others. Occasionally the final results are apparently contradictory.

Taken together, if in a given case of deafness Weber's test and mastoid conduction give a positive sign for that side on which Rinné gives a negative one (say the right), we may safely exclude an implication of the right auditory nerve from the diagnosis. If, on the other hand, Rinné gives a positive result with the whole scale of tuning forks in a case in which marked deafness is present, Weber's test and mastoid conduction being negative for the same side, the fault may be assumed to be with the corresponding auditory nerve. For an excellent *résumé* of the whole subject of the various methods of testing the hearing, see Dundas Grant's paper in the Medical Annual for 1894.

